# Higher education expansion and individual health improvement: concurrent discussion of the blocking effect on the intergenerational transmission of health

**DOI:** 10.3389/fpubh.2025.1577096

**Published:** 2025-07-04

**Authors:** Yifei Chu, Sirui Chen, Lixia Zhang

**Affiliations:** ^1^Shanghai Academy of Agricultural Sciences, Shanghai, China; ^2^College of Business, Hunan Agricultural University, Changsha, China

**Keywords:** higher education, individual health, healthy intergenerational transmission, health inequalities, educational expansion

## Abstract

**Background:**

The relationship between higher education and individual health is important for improving regional human capital for health. Considering the differences in education system development and health infrastructure between China and developed countries, it is necessary to explore the specific impact of higher education expansion on individual health in China.

**Methods:**

This study employs the cohort DID approach to examine how higher education expansion affects individual health, with a particular focus on its role in disrupting the intergenerational transmission of health, drawing data from the China Education Statistical Yearbook and the China Health and Retirement Longitudinal Study (CHARLS).

**Results:**

Results show that higher education expansion promotes individual health improvements by enhancing academic qualifications and access to better employment opportunities, with urban dwellers, women and individuals from well-educated families receiving more benefits. Higher education expansion disrupts the intergenerational transmission of health, and younger individuals exposed to new academic and social environments tend to develop health-related beliefs and behaviors that differ from their family influences.

**Conclusion:**

This study provides insight into the social benefits of higher education, and furthermore offers a new perspective on addressing long-standing health inequalities.

## 1 Introduction

Higher education has grown rapidly throughout the world in the 21st century (1–3). In addition to developed and wealthy industrialized countries, developing countries are also expanding higher education, in which China is at the forefront (4, 5). The Chinese government has invested heavily in expanding access to higher education, resulting in significant increases in enrolment and graduation rates in recent decades (6, 7). The expansion of higher education has had far-reaching economic and social impacts, including economic growth, productivity gains, labor markets, educational equity, social welfare and happiness (7–11).

The positive correlation between education and health is well established (12–16). Well-educated individuals often correlate with higher social status, better occupations, and higher incomes, which are also associated with a better state of health (17–20). Theoretically, when controlling for other factors, those with higher educational attainment tend to achieve greater health returns on their investments in health capital (21–23). Despite these well-established links, the education system and health infrastructure in developing countries can differ significantly from those in developed countries. Therefore, it is essential to further explore the specific impacts of expanding higher education in these contexts. Especially, with accelerating global aging and declining fertility rates, understanding the relationship between higher education and individual health becomes increasingly crucial for enhancing health human capital in deprived areas. In addition, given the prevalence of intergenerational health transmission, examining whether the significant impact of higher education expansion on individual health disrupts this transmission—enabling group health to converge toward an average level—has important implications for health equity research.

In recent decades, China’s higher education system has expanded dramatically. The number of university graduates has risen from less than 1 million in 1990 to over 9 million in 2020 (24), representing one of the most significant investments in higher education among middle-income countries (25). The massive expansion of higher education in China provides a unique and more representative natural experimental context for studying the relationship between higher education and health in developing countries and for exploring how changes in educational opportunities affect the intergenerational transmission of health within families.

This study aims to address two key questions: Does the expansion of higher education contribute to individual health improvements? Since individual health is often linked to the health of other family members (e.g., intergenerational transmission of health), whether this policy would reduce health inequalities by blocking the intergenerational transmission of health? The results have implications not only for understanding the wider societal benefits of education, but also for the development of policies aimed at reducing health inequalities.

This study contributes to the literature in three ways. First, it links the expansion of higher education to individual health outcomes. Existing research has extensively documented the positive relationship between education and health in developed regions such as the United States, Italy and Germany (26–28). However, studies explicitly examine the causal impact of large-scale higher education policies in low-and middle-income countries (e.g., China’s 1999 expansion) on individual health are limited. Using a cohort DID approach, this study provides robust evidence on how increased access to higher education affects self-reported health outcomes in the context of rapid development. Like this study, Fu et al. (29) used the 2SLS approach to estimate the impact of higher education expansion on individual health in China, which provides good insights. Differently, this paper uses a cohort DID model to improve the precision of the estimation and discuss the role of higher education expansion in attenuating the intergenerational transmission effect of health.

Second, this study provides an in-depth discussion of the social and economic consequences of China’s higher education expansion policies. China’s 1999 higher education expansion has been widely studied for its profound socio-economic impacts, such as increasing educational attainment, enhancing income mobility, increasing unemployment, and exacerbating social stratification (6, 20, 25, 30–32). However, its implications for health improvement remain underexplored. By focusing on individual health as a critical outcome of educational policies, this study complements existing research by emphasizing the broader societal benefits of education. Specifically, this study examines how increased access to higher education influences health outcomes and reduces health disparities. This perspective highlights the interconnections of education, health, and socio-economic well-being, offering a more comprehensive understanding of the long-term effects of education policy.

Third, this paper contributes to the literature on breaking the intergenerational transmission of health through education. While the literature has clearly identified the existence of intergenerational transmission effects on health (33–37), there has been limited focus on identifying effective strategies to interrupt or mitigate this transmission relationship. This is particularly important because the continuation of poor health outcomes across generations can perpetuate cycles of disadvantage and inequality. In this context, this study explores the role of higher education expansion as a potential mechanism for disrupting this cycle. This study therefore provides valuable insights into how educational policy interventions can serve as a powerful tool for breaking the intergenerational link between education, health, and socio-economic outcomes.

The paper is structured as follows. Section 2 describes China’s higher education expansion policy. Section 3 presents the data and the empirical strategy. Section 4 discusses the empirical results, including the main results, robustness tests and heterogeneity analysis. Section 5 discusses the mechanisms by which higher education expansion affects health and the blocking effect of higher education expansion on the intergenerational transmission of health. Section 6 concludes the study.

## 2 Materials and methods

### 2.1 Institutional background

#### 2.1.1 Expansion of higher education

Since the reform and opening in 1978, the development of higher education in China can be roughly divided into two phases. Before 1999, the gross enrolment rate in higher education basically remained at a low level, with only 2.4% of young people aged 18–22 being able to enter higher education. Even with the addition of the Adult College Entrance Examination, the rate was only 4%. According to Trow (38), a gross enrolment rate of less than 15% in higher education is considered to be elite education, which means that higher education in China was still in the hands of an elite minority until 1998.

After more than a decade of rapid development under reform and opening up, a number of socio-economic problems have emerged in China, such as weak economic growth and rising unemployment. On the economic front, China’s economy overheated in the early 1990s, with the overall CPI reaching 124.1 in 1994. Chinese policymakers adopted a series of economic cooling measures and succeeded in lowering the inflation level, with the overall CPI falling to 102.8 in 1997. However, China’s economic growth has also slowed down rapidly, declining from over 14% to less than 8% for six consecutive years since 1993.^1^ In terms of employment, the number of people employed in China’s state-owned enterprises (SOEs) has declined year on year since 1995 (39). In 1998, employment in SOEs was down by 1.86 million from 1997, and China began to experience a “wave of layoffs” (40).

According to the Monitoring Center for Economic Prosperity, about 80% of China’s urban residents subscribe to the view that ‘no matter how poor you are, you cannot afford to be poor in education.’ Education is regarded as a key measure for maintaining social stability, easing employment pressure, expanding domestic demand and stimulating economic growth. Against this background, China launched a higher education development program in 1999 and issued the “Action Plan for the Revitalization of Education for the 21st Century.” According to this document, China’s higher education enrolment rate should be close to 15% by 2010. Since then, China’s higher education has entered the second stage of rapid development.

#### 2.1.2 Characterization facts

The policy of expanding higher education has brought about far-reaching changes in China.

**Figure 1:**
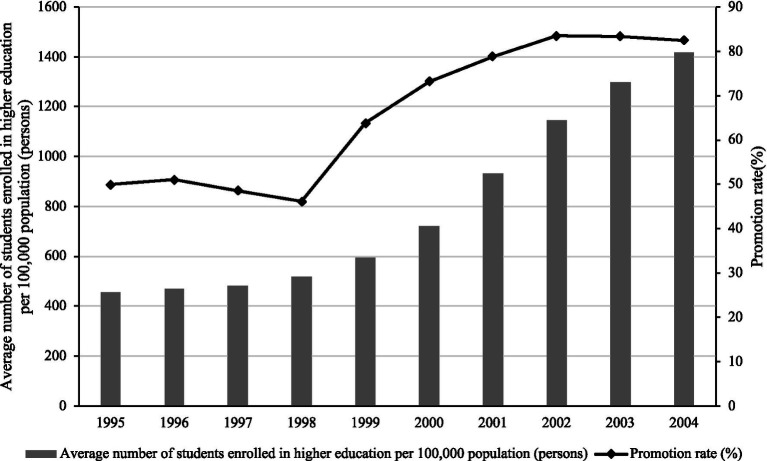
Admission to higher education in China 1995–2005. Data source: China Education Statistical Yearbook. Promotion rate of senior secondary school graduates is the ratio of total number of new entrants admitted to Higher Education Institutions to the total number of graduates of regular senior secondary schools of the current year.

First, the number of people receiving higher education in China has increased dramatically, with the system of higher education gradually transitioning from elite education to universal education. This study calculated the average number of students enrolled in higher education and promotion rate per 100,000 people for the period 1995–2004 (Figure 1). As shown in the figure, the rate of progression to upper secondary school jumped considerably between 1998 and 1999 (from 46.1 to 63.8%). Correspondingly, the average number of students enrolled in higher education per 100,000 populations also rose substantially (from 519 to 594). Since 2002, the promotion rate has exceeded 80%, meaning that more than 80 out of every 100 senior high school graduates in China now continue to higher education. By 2004, the average number of students enrolled in higher education per 100,000 population surpassed 1,400, approaching the level of developed countries such as the United Kingdom.

Second, the gap in higher education resources between different regions has widened. This study compared the differences in undergraduate and specialized enrolment in higher education resources across 31 provinces and cities in China both before the expansion of colleges and universities in 1998 and after the implementation of the university expansion policy in 1999, as shown in Table 1. As illustrated in the table, the number of students enrolled in China’s higher education institutions increased by 657,518 in 1999, with more than 40% of the expansion concentrated in six provinces—Shandong, Hubei, Jiangsu, Henan, Hebei, and Guangdong—which also had the largest enrolments in 1998. This suggests that the implementation of the university expansion policy varied significantly across provinces. Provinces with more substantial higher education resources tended to enroll more students in this round of expansion, thereby expanding the size of their institutions and reinforcing their advantages in higher education.

**Table 1:** Enrolment in higher education in each province (1998, 1999).

Province	1998 enrolment	1999 enrolment	Expansion
Total	1,548,554	2,206,072	657,518
Shandong	82,410	135,185	52,775
Hubei	96,375	142,237	45,862
Jiangsu	127,013	172,491	45,478
Henan	78,805	122,896	44,091
Hebei	72,856	111,033	38,177
Guangdong	85,341	123,338	37,997
Sichuan	65,481	100,965	35,484
Shaanxi	68,034	97,209	29,175
Anhui	51,726	80,204	28,478
Hunan	77,237	105,237	28,000
Liaoning	87,851	113,319	25,468
Zhejiang	52,657	77,608	24,951
Beijing	78,429	97,736	19,307
Shanghai	63,244	81,328	18,084
Heilongjiang	62,480	79,970	17,490
Guangxi	32,596	49,432	16,836
Chongqing	34,374	49,951	15,577
Jilin	52,553	67,807	15,254
Inner Mongolia	18,253	33,456	15,203
Fujian	38,710	53,267	14,557
Jiangxi	43,586	57,921	14,335
Xinjiang	19,435	33,280	13,845
Tianjin	31,670	45,468	13,798
Shanxi	37,897	49,428	11,531
Gansu	23,010	33,825	10,815
Guizhou	24,810	33,718	8,908
Yunnan	27,502	33,879	6,377
Hainan	4,903	8,181	3,278
Qinghai	3,172	6,176	3,004
Ningxia	4,487	7,207	2,720
Tibet	1,657	2,320	663

Source: China Education Statistical Yearbook.

The university admission system in China follows the principle of territorial, with local colleges and universities favoring candidates from their province. Thus, the richer the educational resources of a province, the larger the scale of the expansion, and the higher the likelihood that candidates from that province will be admitted to universities. Existing studies have shown that the uneven spatial distribution of higher education resources is a key factor explaining why high school graduates from different regions are affected differently by the expansion policy (41). The regional disparities in how expansion policies affect young people provide a practical foundation for constructing the cohort DID model.

#### 2.1.3 Education and health

Numerous health studies have shown that the length of education is an important factor in health levels. This always holds regardless of the measure of health (mortality, morbidity or days of work lost) and whether individual data or overall averages are used (42–47). Grossman’s Health Production Function framework (1972) provides theoretical support for this view. The more educated people are assumed to be more effective health producers, they have fewer unhealthy habits and go to the doctor when needed. That is, higher income not only provides individuals with more resources to invest in their health but also increases their demand for higher-quality healthcare and services. Moreover, increased educational attainment enhances an individual’s efficiency in “producing” health, enabling consumers to make more effective use of limited resources to maintain and improve their health. The policy of expanding access to university education plays a crucial role within this theoretical framework. It not only significantly boosts an individual’s level of education and broadens their intellectual horizons, but also improves employment prospects and income levels. In doing so, it provides a stronger economic foundation and informational support for health investments.

**Figure 2:**
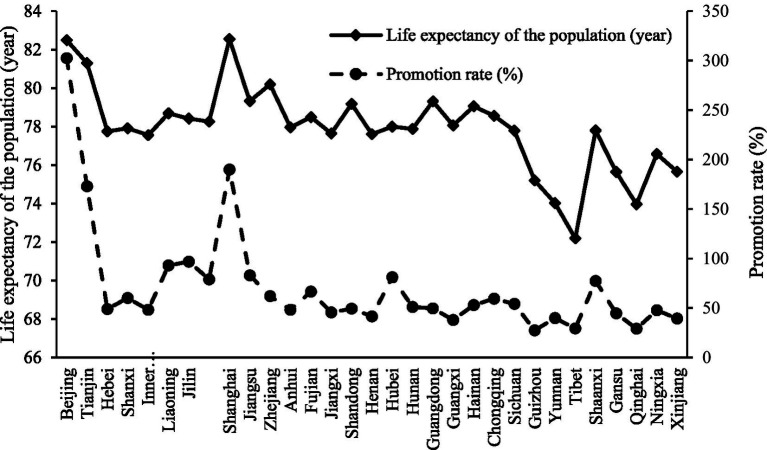
Relationship between life expectancy and promotion rate in each province. Data source: China Education Statistical Yearbook; China Health Statistics Yearbook. Life expectancy does not distinguish between years lived in good health and years lived with disability or chronic illness; healthy life expectancy would more accurately capture the relationship between regional higher-education expansion and population health. However, we currently lack provincial-level healthy life expectancy data, which represents one of the limitations of this study.

To further examine the relationship between higher education resources and health outcomes, this study analyzed data from 2020 for each province, focusing on higher education resources and life expectancy per capita (Figure 2). The results show that provinces with abundant higher education resources tend to have higher life expectancy per capita, suggesting that access to higher education resources may play an important role in improving population health.

Based on theoretical analyses and macro-statistical evidence, this study believes that there is a certain positive relationship between higher education expansion and individual health. The following hypothesis is proposed:

*Hypothesis* 1 Expansion of higher education significantly improves the health status of individuals.

Building on Hypothesis 1, we now broaden our focus to examine the crucial role of higher education in disrupting the intergenerational transmission of health. Intergenerational health transmission refers to the multifaceted process through which health conditions, behaviors, and outcomes are passed from parents to their children via genetic inheritance as well as through intertwined social, cultural, and economic channels. In recent years, the significance of these intergenerational processes has garnered considerable scholarly attention. Research employing a range of quantitative health indicators—such as body mass index (BMI) and newborn weight (35)—has revealed a striking and relatively uniform finding: the health status of the paternal generation tends to exert a more pronounced influence on the subsequent generation’s health (48, 49). Since the policies promoting higher education expansion have a significant positive impact on individual health outcomes. This discovery naturally raises an important question: can higher education serve as a mechanism to disrupt, or even sever, the intergenerational transmission of health disparities?

First, most Chinese universities are concentrated in provincial capitals and other economically developed cities, which compels many young people to leave their hometowns and families to pursue higher education. Once immersed in a new learning environment, these individuals are likely to develop health and medical perspectives that differ from the traditional views instilled by their families, thereby diminishing the familial influence on their personal health. Second, the development of higher education increases the human capital of individuals and raises their employment status. Education not only provides important health knowledge and disease prevention skills, but also increases the likelihood that individuals will have access to quality employment opportunities, raise their income levels and improve their material living conditions, thereby mitigating the negative impact of parental health disadvantage on the health of future generations. The following hypothesis is proposed:

*Hypothesis* 2 Expansion of higher education can block intergenerational transmission of health.

### 2.2 Methods

#### 2.2.1 Datasets

This study involves two sets of information: the level of higher education expansion and data on residents. First, this paper collected data on higher education resources across provinces from the China Education Statistical Yearbook and established a provincial dataset. Second, this study matched the province-level information with micro-level survey data. The primary outcome variable was derived from the China Health and Retirement Longitudinal Study (CHARLS), a health survey of Chinese residents.

The CHARLS dataset includes data from 150 district level units and 450 village-level units, covering about 10,000 households. The survey data encompass a wide range of samples from different geographic locations, levels of economic development, and demographic characteristics, making it relatively representative. In terms of research subjects, CHARLS covers middle-aged and older adult individuals aged 45 and above, whose birth years span from 1960 to 1999, as well as their children. This provides a strong sample for studying the impact of the higher education expansion policy that began in 1999 on individuals’ health. CHARLS conducted surveys in 2011, 2013, 2015, 2018, and 2020. Since the 2011 and 2013 surveys did not include questions related to the health of participants’ children, and personal health data in 2020 may have been significantly disrupted by COVID-19, this study used data only from the 2015 and 2018 surveys.

To accurately examine the impact of higher education expansion on individual health, this study applies several sample selection criteria. First, under China’s educational system, adolescents typically take the college entrance examination at the age of 18 or older, making them directly affected by the policy. At the same time, individuals under the age of 18 are still undergoing physical development and experience significant fluctuations in health indicators that hinder accurate assessment. Therefore, samples with offspring aged 18 or above are retained.

Second, the effect of higher education expansion may vary depending on the availability of higher education resources in the region where individuals took the college entrance examination. Since the survey does not directly specify the location of the examination, this study infers this information using the hukou registration and permanent residence of the parent generation. Specifically, this paper only includes samples that the hukou and permanent residence align, using the corresponding province and city as the assumed location of the child’s college entrance examination.

Finally, discrepancies in the dataset are addressed. This study excludes samples with basic personal information such as gender or year of birth that is inconsistent across survey periods, as well as samples with missing or statistically incorrect personal and household variables. After the above screening, a total of 13,403 sample pairs from 24 provinces are obtained. The effective sample size may vary across analyses depending on the variables included and the selected subsamples.

#### 2.2.2 Empirical strategy

This study employs a cohort difference-in-differences (DID) identification strategy leveraging two key sources of variation. First, provinces received different enrollment quotas during the higher education expansion. Second, within the same province, different youth cohorts were affected differently depending on the years of education received. To capture the impact of higher education expansion on individual health improvements, it is essential to accurately identify the groups influenced by this policy, taking into account the unique characteristics of China’s educational system and cohort dynamics.

With 1999 as the cut-off point, access to higher education before that year is categorized as ‘pre-intervention’, while access after 1999 is categorized as ‘post-intervention’. In the Chinese education system, children generally start primary school at the age of 6 and take the college entrance examination at the age of 18. Thus, individuals born in 1981 or later are directly affected by expansion policy.

Following Duflo (50), this paper first estimates the impact of higher education expansion on each cohort separately, using the pre-1981 birth cohort as the baseline:


(1) $$\begin{array}{l} Y\_\mathrm{hea}l_{\text{igpt}}=\beta_{0}+\beta_{1}({\text{cohort}}_{g}\geq 1981)\times \exp_{p}+\beta_{2}X_{\mathrm{ifp}}+\Lambda_{\mathrm{cgt}}+\\ X_{\mathrm{pt}}\times {\text{cohort}}_{g}+\varepsilon_{\text{igpt}} \end{array}$$

The subscript i denotes the individual, p represents the province where the individual resides, g indicates the birth cohort, and t corresponds to the survey year (2015 or 2018). The explanatory variable $Y\_\mathrm{hea}l_{\text{igpt}}$ measures the health level of individual i. The term ${\text{cohort}}_{g}$ captures birth cohort-specific effects, ${\text{cohort}}_{g}\geq 1981$ indicates whether the birth cohort is from 1981 or later, while $\exp_{p}$ represents the magnitude of higher education expansion in province p. $\beta_{1}$ is the correlation parameter to quantify the impact of higher education expansion on individual health level. $X_{i,f,p}$ includes individual, household, and provincial-level control variables such as gender, household income, and per capita fiscal health expenditure.$\;\Lambda_{\mathrm{cgt}}$ denotes a set of fixed effects.$\;X_{\mathrm{pt}}\times {\text{cohort}}_{g}$ denotes interaction terms for province and year variables with birth cohort variables. $\varepsilon_{\text{igpt}}$ is the error term.

First, this study includes year and city fixed effects to control for regional and temporal characteristics that may influence both higher education expansion and individual health but are unrelated to birth cohorts. Second, birth cohort fixed effects account for common characteristics within the same birth year. In addition, the interaction term between survey year and birth cohort variables controls for systematic differences between cohorts due to different survey years. Finally, to account for the effect of initial provincial education levels on the implementation of the higher education expansion policy, this study includes the interaction term between birth cohort and provincial primary education levels prior to the implementation of the policy in 1998. All regressions are clustered at the community/village level, considering any potential correlation of birth cohorts in the same community/village.

#### 2.2.3 Variable settings

The explanatory variable in this study is individual health, measured as self-reported health. Self-reported health is the respondent’s subjective assessment of the general health of their offspring (coded 1 = very poor, 2 = poor, 3 = satisfactory, 4 = good, 5 = very good). Self-reported health is a valid and reliable measure of general physical condition (51, 52).^2^

This study focuses on the regression coefficients of the dummy variable of whether individuals experience higher education expansion (${\text{cohort}}_{g}\geq 1981$) and the interaction term of the degree of expansion ($\exp_{p}$). Individuals born before 1981 are assumed to be unaffected by the higher education expansion policy and are assigned a value of 0. Individuals born in 1981 and later are assumed to be affected by the policy and are assigned a value of 1, resulting in the variable ‘whether they have experienced higher education expansion’. The extent of the expansion is measured by the promotion rate in each province in 1998. Promotion Rate (%) = (Number of Admissions/Number of High School Graduates) * 100.

To avoid poor control (53), ex-ante control variables were selected at the individual, household and district levels. At the individual level, the gender variable ‘Male’ was chosen because there are physiological and pathological differences between men and women, and gender relations continuously have an impact on physical health (54, 55), making it necessary to control for the differential impact of gender on individual health. At the household level, the variable ‘Family income’ was chosen because the structural effects of income on health have been widely demonstrated (56–59). At the same time, paternal age ‘Fat_age’, lifestyle habits ‘Fat_habit’ (never smoked = 0; former or current smoker = 1), and type of occupation ‘Fat_career’ (senior non-manual occupations = 1; general non-manual occupations = 2; industrial and service manual occupations = 3; agricultural manual occupations = 4) were also taken into account. At the district level, the variable ‘Urban’ (urban = 1; rural = 0) and ‘Medical fee’ were selected and ‘district financial inputs for health/resident population’ was used as a measure (60). Descriptive statistics of the relevant variables are shown in Table 2.

**Table 2:** Summary statistics.

Variable	Treatment group (*N* = 7,366)		Control group (*N* = 6,037)	
	Mean	SD	Mean	SD
Health	4.069	0.928	3.661	0.995
Age	29.139	4.453	44.115	5.551
Exp	0.993	0.011	0.991	0.013
Male	0.632	0.482	0.707	0.455
Fat_age	55.266	5.604	69.227	6.150
Fat_career	3.299	0.823	3.561	0.611
Fat_habit	0.395	0.488	0.395	0.489
Family income	5.790	3.618	4.823	3.329
Medical fee	849.971	121.159	852.407	120.935
Urban	0.420	0.493	0.408	0.491

## 3 Results

### 3.1 Baseline regression result

Table 3 show the baseline regression results for the cohort DID estimation. Higher education expansion is statistically significantly correlated with individual health after adjusting for control variables and fixed effects on the different dimensions. As shown in the Column (3) of Table 3, higher education expansion led to a 26.8% improvement in individual health. As can be seen in Table 3, the effect of higher education expansion on individual health level remains robust under different qualifications. The regression results tentatively confirm Hypothesis 1, which states that higher education expansion contributes to the improvement of individual health level.

**Table 3:** Baseline regression results.

Variable	Health		
	(1)	(2)	(3)
Cohort × Exp	0.631***	0.298*	0.268*
	(0.034)	(0.155)	(0.159)
Male			0.089***
			(0.019)
Fat_age			−0.010***
			(0.002)
Fat_career			−0.039***
			(0.013)
Fat_habit			0.008
			(0.017)
Family income			0.007***
			(0.003)
Medical fee			−0.000
			(0.000)
Urban			0.081***
			(0.027)
Cons	3.693***	3.794***	4.461***
	(0.014)	(0.048)	(0.279)
Year FE	√	√	√
City FE	√	√	√
Cohort FE		√	√
Year × Cohort FE		√	√
Base education × Cohort FE		√	√
N	13,403	13,367	13,367
*R* ^2^	0.128	0.206	0.211

Values in parentheses are standard errors clustered by commune. */**/*** indicate statistically significance at the 10%/5%/1%-level. Standard errors are in parentheses and clustered at the county-year level. Part of the sample is absorbed in the regression and the sample size is reduced.

### 3.2 Parallel trends test

Analyses using the cohort difference-in-differences approach must satisfy the parallel trends assumption: if there are no policy shocks to the treatment group, the trends in the outcome variables of the treatment and control groups should be consistent, i.e., not systematically different. In the case of the higher education expansion policy in this paper, if the expansion policy had not been implemented, the trends in health levels of the treatment and control groups should have been consistent at all ages. To this end, this paper follows Duflo (50) in constructing a parallel trend test using a dynamic reduced-form cohort. This study selected the birth cohort that was 18 years old (born in 1980) 1 year before the implementation of the higher education expansion policy as the base group, the sample born before 1980 as the control group, and the birth cohort born after 1980 as the treatment group to identify the average causal effect of the impact of higher education expansion on the health level of the population of a given birth cohort.

**Figure 3:**
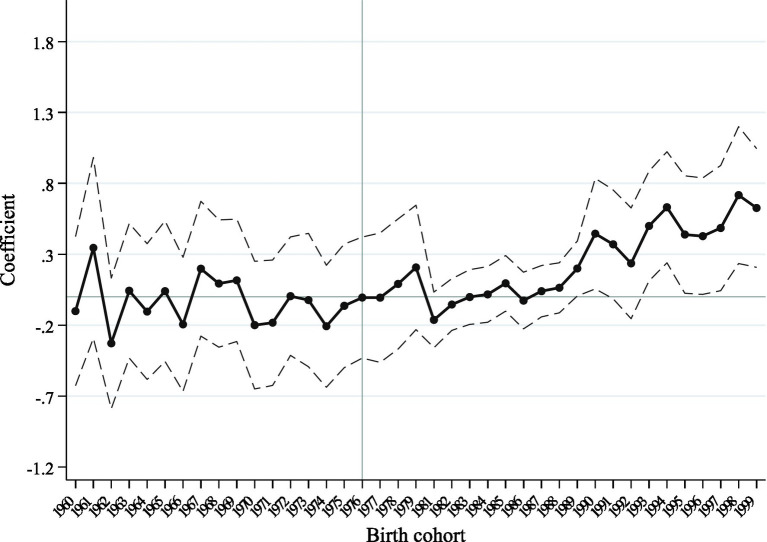
Effect of higher education expansion on the reported-health of different cohorts. Note: Base period for cohorts with birth years prior to 1966.

The results of the parallel trend test on the regression coefficient are shown in Figure 3. For the pre-1980 birth cohort, the estimate of the coefficient does not reject the initial hypothesis of being equal to zero. This indicates that there is no trend of heterogeneity in the individual health levels of different birth years before the start of the higher education expansion policy, and the parallel trend test is passed. For the post-1981 birth cohorts, the 1989 and later birth cohorts begin to be significant and the estimates increase, and most of them pass the significance test, indicating that the higher education expansion policy has begun to promote and continue to enhance individual health levels 10 years after the policy was implemented.

### 3.3 Robustness tests

First, individual health improvement may also be influenced by some random factors. To exclude the influence of random factors on the model, this paper refers to Chen et al. (61) and modifies the placebo test for the control and treatment group design. Assuming the higher education expansion policy was implemented before 1999, the pre-1981 birth cohort (1960–1980) is divided into a control group (1960–1968) and a placebo treatment group (1969–1980), and the regression results are shown in Column (1) of Table 4. The estimates of the impact of the higher education expansion policy on individual health are not statistically significant after changing the time of policy implementation, and the placebo test is passed.

**Table 4:** Robustness test I.

Variable	Health		
	(1)	(2)	(3)
Cohort _1970 × Exp	−0.180		
	(0.280)		
Cohort _1983 × Exp		0.295**	
		(0.139)	
Cohort _1985 × Exp			0.297**
			(0.143)
Control variables	√	√	√
Year FE	√	√	√
City FE	√	√	√
Cohort FE	√	√	√
Year × Cohort FE	√	√	√
Base education × Cohort FE	√	√	√
N	6,018	13,367	13,367
*R* ^2^	0.196	0.212	0.211

Values in parentheses are standard errors clustered by commune. */**/*** indicate statistically significance at the 10%/5%/1%-level. Standard errors are in parentheses and clustered at the county-year level.

An alternative placebo test is to delay the implementation of the policy for several periods, and if the correlation coefficients are strengthened, the policy is proven to be effective. To this end, this paper assumes that higher education expansion policy was implemented in 2001 and 2003, respectively, and reconstructs the ‘Cohort × Exp’ variable to be included in regression model (1). The regression results are presented in Columns (2) and (3) of Table 4. The correlation coefficients are statistically significantly positive and expanding after two and four lags in the implementation of the higher education expansion policy, passing the placebo test.

Second, samples born in 1980, 1981 and 1982 are excluded. Since high school students can postpone the college entrance examination by repeating or enrolling a year earlier, the ‘expansion experience’ variable has a certain self-selection effect. This study excludes all samples born in 1980, 1981 and 1982 because according to the academic system, the samples born in 1980 or 1982 should have taken the university entrance examination in 1998 or 2000, and they would also be directly affected by the expansion of higher education if they repeat a year or enter school a year earlier. The regression results are shown in Column (1) of Table 5. The estimated coefficients are significantly positive at the 10% statistical level and the results are consistent with the benchmark regression.

**Table 5:** Robustness test II.

Variable	Health				
	(1)	(2)	(3)	(4)	(5)
Cohort × Exp	0.262*				0.263*
	(0. 155)				(0.158)
Cohort × Exp2		0.303*			
		(0193)			
Cohort × Exp3			0.001**		
			(0.001)		
Cohort × Exp4				0.002**	
				(0.001)	
Control variables	√	√	√	√	√
Year FE	√	√	√	√	√
City FE	√	√	√	√	√
Cohort FE	√	√	√	√	√
Year × Cohort FE	√	√	√	√	√
Base education × Cohort FE	√	√	√	√	√
N	11,816	13,367	13,367	13,367	12,240
*R* ^2^	0.212	0.213	0.212	0.212	0.209

Values in parentheses are standard errors clustered by commune. */**/*** indicate statistically significance at the 10%/5%/1%-level. Standard errors are in parentheses and clustered at the county-year level.

Third, replace the education resources variable. Considering that there may be errors in the construction of the expansion intensity variable in the baseline regression, this paper reconstructs the measure of the education resources variable. In the regression, the promotion rate is replaced by the ratio of the number of students enrolled in colleges and universities to the number of students enrolled in general high schools in each province and city in 1998. The regression results are shown in column (2) of Table 5. We replace the original ‘promotion rate’ variable with the annual growth in the total number of students enrolled in general undergraduate and specialized colleges and universities in each province from 1998 to 2014 to examine the impact of the scale of expansion on individual health. The regression results are shown in column (3) of Table 5. We also use the annual growth number of enrolments in general undergraduate and specialized colleges and universities in each province from 1998–2014 as a proxy variable for the rate of progression, so as to measure changes in educational resources from the perspective of the scale of expansion of the student population. The regression results are shown in column (4) of Table 5. The results all show that higher education expansion policies continue to significantly improve individual health.

Finally, the influence of compulsory education must be excluded. The introduction of the Compulsory Education Law of the People’s Republic of China in 1986 significantly increased the number of years of education among Chinese residents. In terms of implementation time, the impact group of the 1986 Compulsory Education Law is the post-1980 birth cohort, which overlaps almost exactly with the impact cohort of the higher education expansion policy. In order to exclude spillover effects from compulsory education, this study retains the sample with upper secondary or higher qualifications in the regression. The regression results, as shown in Column (5) of Table 5, indicate that the effect of higher education expansion policy on individual health improvement remains significant. Combined with Huang’s (62) finding that there are no spillover effects from China’s Compulsory Education Law, the expansion of compulsory education does not affect the conclusions of this paper.

### 3.4 Heterogeneity analysis

#### 3.4.1 Urban–rural heterogeneity

**Figure 4:**
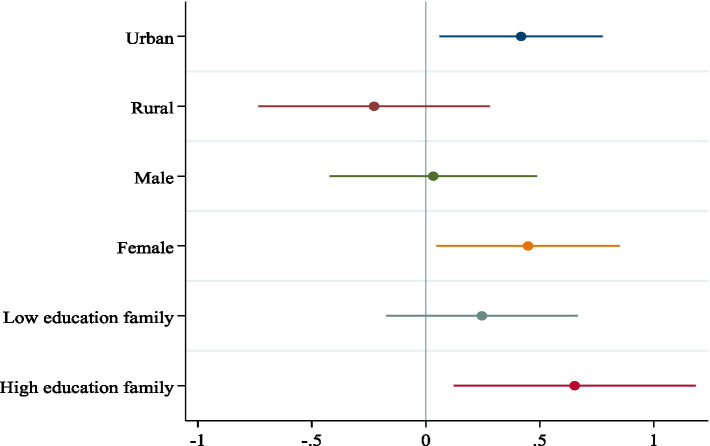
Heterogeneity analysis. This figure shows the estimated coefficients based on Equation 1 using different grouping statistics. Circles indicate point estimates and colored solid lines indicate 90% confidence intervals. Standard errors are in townships.

Secondary education resources are better in urban areas than in rural areas. Due to the differences in basic education resources between urban and rural areas, the impact of the university expansion policy on self-reported health levels may manifest differently. Using the hukou type of the father’s generation as the individual’s hukou type when he/she took the college entrance examination, the sample of respondents is divided into urban and rural samples for the heterogeneity analysis. The regression results are shown in Figure 4. The results show that the higher education expansion policy has a more significant health-promoting effect on urban residents than on rural residents. This may be since the level of primary and secondary education is higher in urban areas, and children from urban families have a greater advantage in the competition for the college entrance examination, and thus benefit more from the promotion of individual health through higher education.

#### 3.4.2 Gender heterogeneity

Under the constraints of Chinese traditional concepts, children of different genders may be tilted by different educational resources in their families (63), which leads to the fact that the effect of higher education expansion policy in promoting individual health can only radiate to a part of the group. This paper examines the gender heterogeneity of the higher education expansion policy in promoting health, and the regression results are shown in Figure 4. Results show that higher education expansion policy significantly improves the health level of women offspring relative to that of men, which corresponds to the reality that women have taken an absolute advantage in the college entrance examination in previous years, and indicates that women are more likely to adopt healthy lifestyles than men after receiving higher education.

#### 3.4.3 Paternal qualifications heterogeneity

Heterogeneity in the educational level of the paternal generation. There is a high correlation between parents’ educational attainment and children’s educational attainment (64–66). The effect of the implementation of the higher education expansion policy may be due to the heterogeneous effect of the different educational levels of the father’s generation. This paper divides respondents’ families into high-education and low-education families based on whether the highest education level of the fathers’ generation is high school or above, and the regression results of the heterogeneous effects of the higher education expansion policy on the health level of individuals in families with different educational levels are shown in Figure 4. The improvement of individual health levels due to higher education expansion is more pronounced in high-education families compared with low-education families.

### 3.5 Mechanical testing

In the theoretical analysis section, we systematically argue that university expansion policies are poised to enhance health outcomes through a dual mechanism—by elevating the probability of individuals gaining access to higher education and by bolstering employment prospects. In the following, this paper uses a two-step regression method to test these two mechanisms of influence.

The first pathway of impact is: improving health by increasing the level of education of individuals. The variable ‘higher education’ is constructed according to the educational level of individuals (college and above = 1; other = 0). This study tests the relationship between the two sets of variables ‘higher education expansion and the probability of an individual’s access to higher education’ and ‘higher education and individuals’ health’ to demonstrate the mechanism that higher education expansion contributes to the improvement of individual health by increasing the probability of individuals’ access to higher education. The results show that the probability of an individual obtaining a university education increases significantly by 17.6% after higher education expansion. After including the mediator variable Edu_collage, the effect of the explanatory variable Cohort × Exp on Health becomes insignificant, while Edu_collage has a positive effect on Health at the 1% significance level, indicating that Edu_collage exerts a full mediating effect. This proves the mechanism by which higher education expansion improves individual’s health by increasing the level of education.

The second influence pathway is: improving health through individual improvement of employment status. Li and Zhu (67) have classified occupations into four main categories: senior non-manual occupations, general non-manual occupations, industrial and service manual occupations, and agricultural manual occupations, respectively. As China’s occupational system is not yet fully defined, it is appropriate to classify of occupations as coarse rather than fine (68–70). This paper divides individual occupations into two categories according to ‘individual occupations’ (non-manual labor occupations = 1; manual labor occupations = 0).^3^ By testing the relationship between the two groups of variables ‘higher education expansion and individual occupations’ and ‘individual occupations and individual health’, this study proves that higher education expansion promotes individual health improvement by increasing individual non-manual labor occupations. The regression results are presented in Columns (3) and (4) of Table 6. The results show that the probability of an individual obtaining a better job increased significantly by 15.9% after the expansion of higher education. After the inclusion of the mediator variable Career_high, the effect of the explanatory variable Cohort × Exp on health became insignificant, whereas Career_high had a positive effect on health at the 1% level of significance, which suggests that Career_high played a full mediating effect. This proves the mechanism by which higher education expansion improves individual’s health by improving individual’s employment status.

**Table 6:** Mechanical testing.

Variable	Edu_collage	Health	Career_high	Health
	(1)	(2)	(3)	(4)
Cohort × Exp	0.176*	0.218	0.159*	0.234
	(0.090)	(0.159)	(0.088)	(0.157)
Edu_collage		0.285***		
		(0.023)		
Career_high				0.214***
				(0.021)
Control variables	√	√	√	√
Year FE	√	√	√	√
City FE	√	√	√	√
Cohort FE	√	√	√	√
Year × Cohort FE	√	√	√	√
Base education × Cohort FE	√	√	√	√
N	13,367	13,367	13,367	13,367
*R* ^2^	0.274	0.222	0.209	0.230

Values in parentheses are standard errors clustered by commune. */**/*** indicate statistically significance at the 10%/5%/1%-level. Standard errors are in parentheses and clustered at the county-year level.

### 3.6 Interruption of intergenerational transmission of health

First, this paper clarifies that there is a high correlation between the health levels of older and younger people in the family. This study counts the distribution of paternal health at different levels of health in the offspring (Table 7). The meaning of the data in the table is that when the health level of offspring is ‘1 (Very Bad)’, the probability that the health level of parent is ‘1’ is 15.7%. Statistically, the probability of the offspring’s health has the same score as the health of the parent is the highest in the cohort comparisons.

**Table 7:** Relationship between offspring health and paternal health.

Variable		Self-reported Health_Father				
		1	2	3	4	5
Self-reported Health_Son	1	0.157	0.339	0.405	0.033	0.066
	2	0.115	0.390	0.377	0.066	0.053
	3	0.071	0.249	0.561	0.067	0.053
	4	0.043	0.170	0.512	0.195	0.080
	5	0.030	0.124	0.478	0.140	0.228

Second, this study constructs appropriate econometric models to test whether higher education expansion blocks this intergenerational transmission of health. This study constructs a variable to identify mobility in health levels: taking the mean of paternal health and the mean of offspring health for the same age and township as a benchmark, and calculating the difference between the parent’s health level and the corresponding mean (as well as the offspring’s health level). A larger difference represents a more favorable health level for the sample in its age group. The difference of the parent generation is subtracted from the difference of the offspring, when the difference is greater than 0, it represents a downward flow of health. When the difference is equal to 0, it represents no flow of health. When the difference is less than 0, it represents an upward flow of health.

After clarifying the flow of health levels, this study constructs the model (2):


(2) $$\begin{array}{l} \text{Healt}h_{\_}\text{Mobillit}y_{\text{igpt}}=\gamma_{0}+\gamma_{1}\mathrm{Fa}t_{\_}\text{Healt}h_{\text{igpt}}+\gamma_{2}(\begin{array}{l} {\text{cohort}}_{g}\geq \\ 1981 \end{array})\times \\ \exp_{p}+\gamma_{3}({\text{cohort}}_{g}\geq 1981)\times \exp_{p}\times \\ \mathrm{Fa}t_{\_}\text{Healt}h_{\text{igpt}}+\beta_{2}X_{\mathrm{ifp}}+\Lambda_{\mathrm{cgt}}+\\ X_{\mathrm{pt}}\times {\text{cohort}}_{g}+\varepsilon_{\text{igpt}} \end{array}$$

In Equation (2), $\mathrm{Fa}t_{\_}\text{Healt}h_{\text{igpt}}$ represents the health level of the parent generation and $({\text{cohort}}_{g}\geq 1981)\times \exp_{p}\times \mathrm{Fa}t_{\_}\text{Healt}h_{\text{igpt}}$ represents the cross term between the health level of the parent generation and the expansion of higher education. Other variables are the same meaning as in model (1). The coefficient $\gamma_{3}$ is of interest.

Table 8 shows the estimated coefficient of the cross term between higher education expansion and paternal health level is negative and significantly not equal to 0 in the analyses of upward intergenerational health mobility, indicating that the higher the health level of the father’s generation, the lower the probability of upward mobility of the offspring’s health. However, in the analyses of downward intergenerational health mobility, the estimated coefficient of the cross term between higher education expansion and paternal health level is significantly positive, indicating that the higher the paternal health level, the greater the probability of downward intergenerational health mobility. In other words, the lower the health level of paternal generation, the greater the probability of intergenerational health flowing upward. In combination, the health of the offspring regresses toward the mean, which is consistent with the findings of Galton (71, 72). The intergenerational transmission of health is blocked and intergenerational health tends to be equitable.

**Table 8:** Higher education and the intergenerational transmission of blocked health.

Variable	up_flow	up_flow	up_flow	down_flow	down_flow	down_flow
		Rural	Urban		Rural	Urban
	(1)	(2)	(3)	(4)	(5)	(6)
Fat_Health	−0.125***	−0.131***	−0.095***	0.125***	0.131***	0.102***
	(0.006)	(0.007)	(0.015)	(0.006)	(0.007)	(0.017)
Cohort × Exp	0.040	0.001	0.184	−0.232***	−0.355***	−0.157
	(0.090)	(0.143)	(0.118)	(0.072)	(0.137)	(0.120)
Fat_Health × Cohort × Exp	−0.036***	−0.026	−0.084***	0.023*	0.030*	0.031
	(0.013)	(0.016)	(0.028)	(0.014)	(0.016)	(0.030)
Control variables	√	√	√	√	√	√
Year FE	√	√	√	√	√	√
City FE	√	√	√	√	√	√
Cohort FE	√	√	√	√	√	√
Year × Cohort FE	√	√	√	√	√	√
Base education × Cohort FE	√	√	√	√	√	√
N	13,367	10,727	2,476	13,367	10,727	2,476
*R* ^2^	0.150	0.159	0.284	0.138	0.157	0.283

Values in parentheses are standard errors clustered by commune. */**/*** indicate statistically significance at the 10%/5%/1%-level. Standard errors are in parentheses and clustered at the county-year level.

## 4 Discussion

This study explores the impact of higher education expansion on individual health, revealing a significant positive relationship. The findings highlight two primary mechanisms by which higher education expansion promotes health improvement: enhanced qualifications and access to better employment opportunities.

First, the expansion of higher education has substantially increased the likelihood of individuals obtaining advanced qualifications compared to the pre-expansion period. This access to higher education directly improves health by enhancing knowledge, behaviors, and access to health-related resources. Second, the expansion has facilitated transitions into non-manual labor and higher-quality jobs. Improved working conditions reduce physical strain and contribute to better health outcomes.

However, the health benefits of higher education expansion are not distributed equally. Urban residents, women, and individuals from well-educated families experience more pronounced improvements in health, underscoring persistent disparities in the accessibility and impact of higher education. Moreover, this study identifies an unintended effect: higher education expansion interrupts the intergenerational transmission of health. Young individuals exposed to new academic and social environments tend to develop health-related beliefs and behaviors that diverge from their familial influences.

The robust association between higher educational attainment and improved health outcomes—documented by Cutler and Lleras-Muney (73) and further corroborated in our analysis—underscores the imperative for targeted, evidence-driven interventions at the provincial level. Given that the recent expansion of higher education in China has demonstrably increased access to non-manual employment and generated larger health returns for rural residents, women, and those from well-educated families, we propose a five-point policy framework designed to amplify these benefits, foster equity, and facilitate rigorous evaluation:

(1)  Equitable Access through Rural and Low-Income Quotas. To maximize health dividends among underserved populations, provincial governments should reserve a designated share of newly created university seats for students from rural or economically disadvantaged backgrounds. Building on the success of similar quota systems in India and Brazil, this measure would directly channel educational opportunities to those who, per our findings, experience the greatest health uplift per additional year of schooling (74).
(2)  Gender-Sensitive Scholarship and Mentorship Programs. Recognizing that female students reap particularly large health benefits from higher education, we recommend the establishment of merit-and need-based scholarships exclusively for young women, coupled with mentorship networks linking them to female professionals in health-promoting industries. Such programs should include leadership workshops, career counseling, and financial support for skill-building activities, thereby narrowing both educational and health-outcome gaps.
(3)  Strengthening Industry–University Pipelines. To ensure that graduate’s transition into healthier, less physically taxing occupations, universities should formalize partnerships with employers in the service, technology, and finance sectors. These partnerships would guarantee internships and placement offers tied to ergonomic workplaces and comprehensive occupational-health standards, in line with ILO guidelines. By smoothing the school-to-work pathway, this policy addresses our result that non-manual labor mediates a significant portion of education’s health effect.
(4)  Two-Generation Upskilling and Lifelong Learning Incentives. Our evidence on intergenerational transmission of health advantages motivates a dual-generation approach: alongside student quotas, local governments should subsidize adult-education and vocational training programs for parents in disadvantaged families. Moreover, tax credits for employers who support continuing-education courses would reinforce healthy trajectories across the lifespan, ensuring that both current students and their families benefit from enhanced human capital.
(5)  Comprehensive Monitoring and Evaluation Framework. Finally, we call for the routine collection and public dissemination of both educational and health indicators at the subnational level—including self-rated health, key biomarkers, and labor-market outcomes—to assess the real-time impact of these policies. Leveraging China’s existing statistical infrastructure and integrating small-area HALE estimation methods will permit policymakers to iteratively refine quota allocations, scholarship targets, and industry partnerships, thereby sustaining and scaling the health gains of higher education expansion.

In conclusion, the expansion of higher education serves as a powerful tool for improving individual health. However, to maximize its benefits, policymakers and educators need to address the inequities and unintended consequences identified in this study. By fostering inclusive and health-oriented educational strategies, higher education can contribute more effectively to societal well-being and sustainable development.

## Data Availability

The raw data supporting the conclusions of this article will be made available by the authors, without undue reservation.

## Footnotes

^1^Data source: China Statistical Yearbook.

^2^Self-reported health may be biased by individual perceptions, cultural background, and social expectations, and we acknowledge this as a key limitation of our study.

^3^‘Non-manual labor’ includes “heads of State organs, party organizations, enterprises and institutions; professional and technical personnel; clerical and related personnel,” and “manual labor” includes “commercial and service workers; agricultural, forestry, fishery and water conservancy workers; and operators of production and transport equipment and related personnel.” ‘Manual labor’ includes workers in the commercial and service industries; workers in the agricultural, forestry, fisheries and water conservancy industries; and operators of production and transport equipment and related personnel.

## References

[1] Cantwell B Marginson S Smolentseva A. High participation systems of higher education. Oxford: Oxford Academic (2018).

[2] Freeman RB. What does global expansion of higher education mean for the US. Cambridge, MA: National Bureau of Economic Research (2009).

[3] Schofer E Meyer JW. The worldwide expansion of higher education in the twentieth century. Am Sociol Rev. (2005) 70:898–920. doi: 10.1177/000312240507000602

[4] Mlambo M Silén C McGrath C. Lifelong learning and nurses' continuing professional development, a metasynthesis of the literature. BMC Nurs. (2021) 20:62. doi: 10.1186/s12912-021-00579-2, PMID: 33853599 PMC8045269

[5] Polat S. The expansion of higher education in Turkey: access, equality and regional returns to education. Struct Chang Econ Dyn. (2017) 43:1–14. doi: 10.1016/j.strueco.2017.06.001

[6] Li S Whalley J Xing C. China's higher education expansion and unemployment of college graduates. China Econ Rev. (2014) 30:567–82. doi: 10.1016/j.chieco.2013.08.002

[7] Ou D Zhao Z. Higher education expansion in China, 1999–2003: impact on graduate employability. China World Econ. (2022) 30:117–41. doi: 10.1111/cwe.12412

[8] Jang E. When education is positional: higher education expansion, welfare regimes and income inequality. J Soc Policy. (2024) 54:1–22. doi: 10.1017/S0047279424000151, PMID: 40395388

[9] Jaume D. The labor market effects of an educational expansion. J Dev Econ. (2021) 149:102619. doi: 10.1016/j.jdeveco.2020.102619

[10] Nikolaev B. Does higher education increase hedonic and eudaimonic happiness. J Happiness Stud. (2018) 19:483–504. doi: 10.1007/s10902-016-9833-y

[11] Yao Y. Does higher education expansion enhance productivity. J Macroecon. (2019) 59:169–94. doi: 10.1016/j.jmacro.2018.11.009

[12] Eide ER Showalter MH. Estimating the relation between health and education: what do we know and what do we need to know. Econ Educ Rev. (2011) 30:778–91. doi: 10.1016/j.econedurev.2011.03.009

[13] Groot W Brink HM. The health effects of education. Econ Educ Rev. (2007) 26:186–200. doi: 10.1016/j.econedurev.2005.09.002

[14] Hartog J Oosterbeek H. Health, wealth and happiness: why pursue a higher education. Econ Educ Rev. (1998) 17:245–56.

[15] Raghupathi V Raghupathi W. The influence of education on health: an empirical assessment of OECD countries for the period 1995–2015. Arch Public Health. (2020) 78:20. doi: 10.1186/s13690-020-00402-5, PMID: 32280462 PMC7133023

[16] Ross CE Wu CL. The links between education and health. Am Sociol Rev. (1995) 60:719–45.

[17] Jehn A. The relationship between postsecondary education and adult health behaviors. SSM-Population Health. (2022) 17:100992. doi: 10.1016/j.ssmph.2021.100992, PMID: 35036513 PMC8749134

[18] Sewell WH Hauser RM. Causes and consequences of higher education: models of the status attainment process. Am J Agric Econ. (1972) 54:851–61.

[19] Wilson OWA Matthews PJ Duffey M Papalia Z Bopp M. Changes in health behaviors and outcomes following graduation from higher education. Int J Exerc Sci. (2020) 13:131–9. doi: 10.70252/TDLP9874, PMID: 32148637 PMC7039470

[20] Xing Y Hu Y Zhou J. Higher education and family background: which really matters to individual’s socioeconomic status development in China. Int J Educ Dev. (2021) 81:102334. doi: 10.1016/j.ijedudev.2020.102334

[21] Berger MC Leigh JP. Schooling, self-selection, and health. Hum Resour Manag J. (1989) 24:433–55.

[22] Goldman DP Lakdawalla DN. Understanding health disparities across education groups. Cambridge, MA: National Bureau of Economic Research (2001).

[23] Grossman M. The demand for health: A theoretical and empirical investigation. NY: Columbia University Press (1972).

[24] UNESCO. (2020). Global education monitoring report, 2020: Inclusion and education: All means all. 3rd edition. Paris: UNESCO.

[25] Huang B Tani M Wei Y Zhu Y. Returns to education in China: evidence from the great higher education expansion. China Econ Rev. (2022) 74:101804. doi: 10.1016/j.chieco.2022.101804

[26] Bratti M Cottini E Ghinetti P. Education, health and health-related behaviors: evidence from higher education expansion. Rochester, NY: Social Science Research Network (2022).

[27] Frase RT Bauldry S. The expansion of higher education and the education-health gradient in the United States. Soc Curr. (2021) 9:70–86. doi: 10.1177/23294965211021645

[28] Jürges H Reinhold S Salm M. Does schooling affect health behavior? Evidence from the educational expansion in western Germany. Econ Educ Rev. (2011) 30:862–72. doi: 10.1016/j.econedurev.2011.04.002

[29] Fu H Ge R Huang J Shi X. The effect of education on health and health behaviors: evidence from the college enrollment expansion in China. China Econ Rev. (2022) 72:101768. doi: 10.1016/j.chieco.2022.101768

[30] Che Y Zhang L. Human capital, technology adoption and firm performance: impacts of China’s higher education expansion in the late 1990s. Econ J. (2018) 128:2282–320. doi: 10.1111/ecoj.12524

[31] Ou D Hou Y. Bigger pie, bigger slice? The impact of higher education expansion on educational opportunity in China. Res High Educ. (2018) 60:358–91. doi: 10.1007/s11162-018-9514-2

[32] Wang X Liu J. China’s higher education expansion and the task of economic revitalization. High Educ. (2011) 62:213–29. doi: 10.1007/s10734-010-9383-x

[33] Ahlburg DA. Intergenerational transmission of health. Am Econ Rev. (1998) 88:265–70.

[34] Bencsik P Halliday TJ Mazumder B. The intergenerational transmission of mental and physical health in the United Kingdom. J Health Econ. (2023) 92:102805. doi: 10.1016/j.jhealeco.2023.102805, PMID: 37804551

[35] Dolton PJ Xiao M. The intergenerational transmission of BMI in China. Econ Hum Biol. (2015) 19:90–113. doi: 10.1016/j.ehb.2015.06.002, PMID: 26398848

[36] Lundborg P Nordin M Rooth DO. The intergenerational transmission of human capital: the role of skills and health. J Popul Econ. (2018) 31:1035–65. doi: 10.1007/s00148-018-0702-3

[37] Thompson O. Genetic mechanisms in the intergenerational transmission of health. J Health Econ. (2014) 35:132–46. doi: 10.1016/j.jhealeco.2014.02.003, PMID: 24674912

[38] Trow M. Problems in the transition from elite to mass higher education. Berkeley, California: Carnegie Commission on Higher Education (1973).

[39] He H Huang F Liu Z Zhu D. Breaking the ‘iron rice bowl’: evidence of precautionary savings from the Chinese state-owned enterprises reform. J Monet Econ. (2018) 94:94–113. doi: 10.1016/j.jmoneco.2017.12.002

[40] Liu H Zhao Z. Parental job loss and children’s health: ten years after the massive layoff of the SOEs’ workers in China. China Econ Rev. (2014) 31:303–19. doi: 10.1016/j.chieco.2014.10.007

[41] Yao S Wu B Su F Wang J. The impact of higher education expansion on social justice in China: a spatial and inter-temporal analysis. J Contemp China. (2010) 19:837–54. doi: 10.1080/10670564.2010.508586

[42] Buckles K Hagemann A Malamud O Morrill M Wozniak A. The effect of college education on mortality. J Health Econ. (2016) 50:99–114. doi: 10.1016/j.jhealeco.2016.08.002, PMID: 27723470

[43] Cavelaars AE Kunst AE Geurts JJ Crialesi R Grötvedt L Helmert U. Differences in self-reported morbidity by educational level: a comparison of 11 western European countries. J Epidemiol Community Health. (1998) 52:219–27. doi: 10.1136/jech.52.4.219, PMID: 9616407 PMC1756698

[44] Clark D Royer H. The effect of education on adult mortality and health: evidence from Britain. Am Econ Rev. (2013) 103:2087–120. doi: 10.1257/aer.103.6.2087, PMID: 29533059

[45] Meghir C Palme M Simeonova E. Education and mortality: evidence from a social experiment. Am Econ J Appl Econ. (2018) 10:234–56. doi: 10.1257/app.20150365, PMID: 40375338

[46] Miech R Pampel F Kim J Rogers RG. The enduring association between education and mortality: the role of widening and narrowing disparities. Am Sociol Rev. (2011) 76:913–34. doi: 10.1177/0003122411411276, PMID: 26937041 PMC4771063

[47] Stewart WF Ricci JA Chee E Morganstein D Lipton R. Lost productive time and cost due to common pain conditions in the US workforce. JAMA. (2003) 290:2443–54. doi: 10.1001/jama.290.18.2443, PMID: 14612481

[48] Classen TJ Thompson O. Genes and the intergenerational transmission of BMI and obesity. Econ Hum Biol. (2016) 23:121–33. doi: 10.1016/j.ehb.2016.08.001, PMID: 27599025

[49] Coneus K Laucht M Reuss K. The role of parental investments for cognitive and noncognitive skill formation--evidence for the first 11 years of life. Econ Hum Biol. (2012) 10:189–209. doi: 10.1016/j.ehb.2011.01.003, PMID: 21367678

[50] Duflo E. Schooling and labor market consequences of school construction in Indonesia: evidence from an unusual policy experiment. Am Econ Rev. (2001) 91:795–813. doi: 10.1257/aer.91.4.795

[51] Davies AR Ware JE. Measuring health perceptions in the health insurance experiment. Santa Monica, CA: Rand Corporation (1981).

[52] Mossey JM Shapiro E. Self-rated health: a predictor of mortality among the elderly. Am J Public Health. (1982) 72:800–8. doi: 10.2105/ajph.72.8.800, PMID: 7091475 PMC1650365

[53] Angrist JD Pischke J. The credibility revolution in empirical economics: how better research design is taking the con out of econometrics. J Econ Perspect. (2010) 24:3–30. doi: 10.1257/jep.24.2.3

[54] Connell R. Gender, health and theory: conceptualizing the issue, in local and world perspective. Soc Sci Med. (2012) 74:1675–83. doi: 10.1016/j.socscimed.2011.06.006, PMID: 21764489

[55] Verbrugge LM. Gender and health: an update on hypotheses and evidence. J Health Soc Behav. (1985) 26:156–82. doi: 10.2307/2136750, PMID: 3905939

[56] Benzeval M Judge K. Income and health: the time dimension. Soc Sci Med. (2001) 52:1371–90. doi: 10.1016/s0277-9536(00)00244-6, PMID: 11286362

[57] Currie A Shields MA Price SW. The child health/family income gradient: evidence from England. J Health Econ. (2007) 26:213–32. doi: 10.1016/j.jhealeco.2006.08.003, PMID: 16962191

[58] Ettner SL. New evidence on the relationship between income and health. J Health Econ. (1996) 15:67–85. 10157429 10.1016/0167-6296(95)00032-1

[59] Kuehnle D. The causal effect of family income on child health in the U.K. J Health Econ. (2014) 36:137–50. doi: 10.1016/j.jhealeco.2014.03.011, PMID: 24794502

[60] Christopoulos K Eleftheriou K. The fiscal impact of health care expenditure: evidence from the OECD countries. Econ Anal Policy. (2020) 67:195–202. doi: 10.1016/j.eap.2020.07.010

[61] Chen Y Fan ZY Gu XM Zhou LA. Arrival of young talent: the send-down movement and rural education in China. Am Econ Rev. (2020) 110:3393–430. doi: 10.1257/aer.20191414

[62] Huang W. Understanding the effects of education on health: evidence from China. IZA Discussion Paper No. 9225, Institute of Labor Economics (2015).

[63] Yang J Huang X Liu X. An analysis of education inequality in China. Int J Educ Dev. (2014) 37:2–10. doi: 10.1016/j.ijedudev.2014.03.002

[64] Burger K. Intergenerational transmission of education in Europe: do more comprehensive education systems reduce social gradients in student achievement. Res Soc Stratification Mobility. (2016) 44:54–67. doi: 10.1016/j.rssm.2016.02.002

[65] Dong Y Luo R Zhang L Liu C Bai Y. Intergenerational transmission of education: the case of rural China. China Econ Rev. (2019) 53:311–23. doi: 10.1016/j.chieco.2018.09.011

[66] Roksa J Potter D. Parenting and academic achievement: intergenerational transmission of educational advantage. Sociol Educ. (2011) 84:299–321. doi: 10.1177/0038040711417013

[67] Li L Zhu B. Intergenerational mobility modes and changes in social class in contemporary China. Soc Sci China. (2017) 38:127–47. doi: 10.1080/02529203.2017.1268380

[68] Lauer C. Education and Unemployment: A French-German Comparison. ZEW Discussion Papers 03-34, Leibniz Centre for European Economic Research. (2003).

[69] Valbuena J. Family background, gender and cohort effects on schooling decisions. Stud Econ Econ. (2011) 6:258–90. Available at: www.kent.ac.uk/economics/repec/1114.pdf

[70] Zou J Deng XJ. Financial literacy, housing value and household financial market participation: evidence from urban China. China Econ Rev. (2019) 55:52–66. doi: 10.1016/j.chieco.2019.03.008

[71] Galton F. Hereditary genius: An inquiry into its laws and consequences. London: Macmillan and Co (1869).

[72] Galton F. Typical Laws of Heredity. Nature. (1877) 15:492–95, 512–14, 532–33.

[73] Cutler DM Lleras-Muney A. Education and health: evaluating theories and evidence. Cambridge: National bureau of economic research working paper, no. 12352 (2006).

[74] Glewwe P Muralidharan K. Improving education outcomes in developing countries: evidence, knowledge gaps, and policy implications. Handbook of the economics of education. (2016) 6:653–743. doi: 10.1016/B978-0-444-63459-7.00010-5

